# Predictors of weaning from helmet CPAP in patients with COVID-19 pneumonia

**DOI:** 10.1186/s13054-021-03627-0

**Published:** 2021-06-12

**Authors:** Dejan Radovanovic, Stefano Pini, Marina Saad, Luca Perotto, Fabio Giuliani, Pierachille Santus

**Affiliations:** Division of Respiratory Diseases, Ospedale Luigi Sacco, Polo Universitario, ASST Fatebenefratelli-Sacco, Department of Biomedical and Clinical Sciences (DIBIC), Università Degli Studi Di Milano, Via G.B. Grassi 74, 20157 Milan, Italy

Continuous positive airway pressure (CPAP) offers a valid non-invasive respiratory support for patients with Coronavirus Disease 2019 (COVID-19) pneumonia [1]. CPAP treatment isn’t free from complications such as pneumothorax/pneumomediastinum, hemodynamic instability, or delirium and requires careful monitoring [1, 2]. Accordingly, timely CPAP removal appears desirable [1, 2]. Our aim was to identify weaning predictors and assess their performance in COVID-19 patients treated with helmet CPAP.

A prospective, observational, cohort study was conducted in our high dependency respiratory unit including consecutive adult patients with laboratory confirmed COVID-19 pneumonia that underwent a weaning trial from CPAP between March 2020 and February 2021 (training cohort).

Patients’ readiness to undergo a weaning trial was judged by the treating physician. A weaning trial was the reduction in support to minimal positive end-expiratory pressure (PEEP≈2 cmH_2_O, including antiviral filters) maintaining a FiO_2_ ≤ 60% [1, 2]. Absence of respiratory distress and SpO_2_ ≥ 94% in the subsequent 30 min lead to helmet removal and oxygen supplementation with FiO_2_ ≤ 60%. A weaning failure was the need to restore CPAP because of respiratory distress or SpO2 ≤ 94% in any moment beginning from the low PEEP trial and during the subsequent 12 h.

Weaning predictors were assessed before reducing PEEP, and included: (1) ROX index (SpO_2_/FiO_2_/respiratory rate (RR)) [3], (2) modified ROX index (partial pressure of oxygen (PaO_2_) to FiO_2_ ratio/RR—mROX) [3], (3) alveolar-arterial (A-a) O_2_ gradient, (4) Sequential Organ Failure Assessment (SOFA) score [4].

Sensitivity and specificity for different thresholds and the area under the receiver operating characteristic curve (AUROC) was calculated for all indexes. The index that best performed in the training cohort was tested in a validation cohort of patients hospitalized in two general wards of our institution. Statistical significance was a p value ≤ 0.05. Analyses were performed with IBM SPSS Statistics V.23.0 (Armonk, NY). The study (NCT04307459) was approved by the local ethical committee (17263/2020) and all patients gave written informed consent.

Seventy-four patients formed the training cohort: 61 (82.5%) succeeded and 13 (17.5%) failed the weaning trial (Table 1). At weaning trial, patients that failed had higher SOFA score, A-a O_2_ and RR, while PaO_2_/FiO_2_, ROX and mROX were higher in patients that succeeded weaning (Table 1). The mROX index had the best AUROC (0.830) and the value that best discriminated weaning success from failure was 8.4 mmHg/bpm (sensitivity 0.80, specificity 0.77) (Fig. 1). This threshold was tested in the validation cohort (44 patients; median age 65, 82% males) of which 32 (72.7%) succeeded and 12 (27.3%) failed weaning. The two cohorts were comparable in terms of clinical characteristics and CPAP duration before weaning. AUROC for mROX in the validation cohort was 0.828, sensitivity and positive predictive value 0.88, specificity and negative predictive value 0.67. Patients with mROX ≥ 8.4 after 5 days of CPAP had twice the probability to be free from CPAP compared with patients with mROX < 8.4 (Fig. 1).

**Table 1:** Clinical characteristics at admission and at weaning trial in patients that succeeded and failed CPAP weaning

Characteristics	Weaning success (n = 61)	Weaning failure (n = 13)	*p* value^a^
Age, years	62 (12)	74 (8)	0.001
Males, n (%)	43 (70)	8 (61)	0.526
Hypertension, n (%)	30 (49)	7 (54)	0.760
Diabetes mellitus, n (%)	13 (21)	3 (23)	0.999
Ischemic heart disease, n (%)	6 (10)	4 (31)	0.067
Obesity, n (%)	26 (43)	6 (46)	0.816
Respiratory disease, n (%)	10 (16)	0 (0)	0.116
CPAP days at weaning trial	4 (2–6)	4 (2.5–5)	0.854
*In-Hospital treatments*			
Antibiotics, n (%)	50 (82.0%)	9 (69.2%)	0.446
LMWH prophylactic, n (%)	39 (63.9%)	8 (61.5%)	0.999
LMWH therapeutic, n (%)	30 (49.2%)	9 (69.2%)	0.189
Systemic corticosteroids, n (%)	46 (75.4%)	9 (69.2%)	0.729
*Clinical status at admission*			
Lymphocytes, × 10^6^/L	900 (600–1400)	800 (700–1000)	0.931
D-Dimer, µg/L FEU	888 (572–2101)	1056 (544–1632)	0.922
CRP, mg/L	85 (42–127)	110 (85–215)	0.060
Creatinine, mg/dL	0.8 (0.7–1.0)	0.9 (0.8–1.6)	0.091
BUN, mg/dL	38 (28–53)	52 (34–70)	0.093
Glasgow coma scale	15 (15–15)	15 (14.5–15)	0.067
SOFA	2 (2–3)	3 (2–4.5)	0.204
Respiratory rate, bpm	24 (22–29)	26 (24–33)	0.275
PaO_2_/FiO_2_, mmHg	194 (122–273)	140 (86.7–281.0)	0.604
A-a O_2_ gradient, mmHg	204 (69–325)	242 (66–336)	0.960
pH	7.48 (0.05)	7.49 (0.05)	0.389
PaCO_2_, mmHg	36 (7)	35 (9)	0.598
ROX index	7.6 (4.8–14.5)	8.1 (4.3–16.3)	0.889
*Clinical status the day of weaning trial*			
D-Dimer, µg/L FEU	899 (545–1425)	1244 (845–1375)	0.183
CRP, mg/L	36 (9–59)	70 (18–115)	0.085
SOFA	2 (1.5 – 3)	3 (3–4)	0.003
GCS	15 (15–15)	15 (15–15)	0.423
A-a O_2_ gradient, mmHg	208 (151–269)	245 (206–445)	0.010
PaO_2_/FiO_2_, mmHg	243 (98)	171 (56)	0.014
Respiratory rate, bpm	20 (18–22)	24 (22–27)	< 0.001
pH	7.45 (7.42–7.47)	7.44 (7.42–7.48)	0.638
PaCO_2_, mmHg	42 (6)	41 (6)	0.653
ROX index	9 (8–11)	7.4 (4.1–8.5)	0.002
mROX index, mmHg/bpm	11.9 (8.5–14.3)	6.6 (5.6–8.8)	< 0.001

Parametric and nonparametric quantitative variables are described with means (standard deviations, SD) and medians (interquartile ranges, IQR), respectively. Chi-squared or Fisher exact test were used to compare qualitative variables, whereas Student t test or Mann–Whitney were used to compare quantitative variables with normal or non-normal distribution, respectively, in patients that failed or succeeded the weaning trial

A-a O_2_ gradient = alveolar-arterial oxygen gradient; BUN = blood urea nitrogen; CPAP = continuous positive airway pressure; CRP = C reactive protein (upper limit of normal 10 mg/L); FEU = fibrinogen equivalent units; GCS = Glasgow Coma Scale; LMWH = low molecular weight heparin; PaO_2_ = arterial partial pressure of oxygen; PaCO_2_ = arterial partial pressure of carbon dioxide; ROX index = SpO_2_/FiO_2_/respiratory rate; mROX index = PaO_2_/FiO_2_/respiratory rate; SOFA = Sequential Organ Failure Assessment

**Fig. 1:**
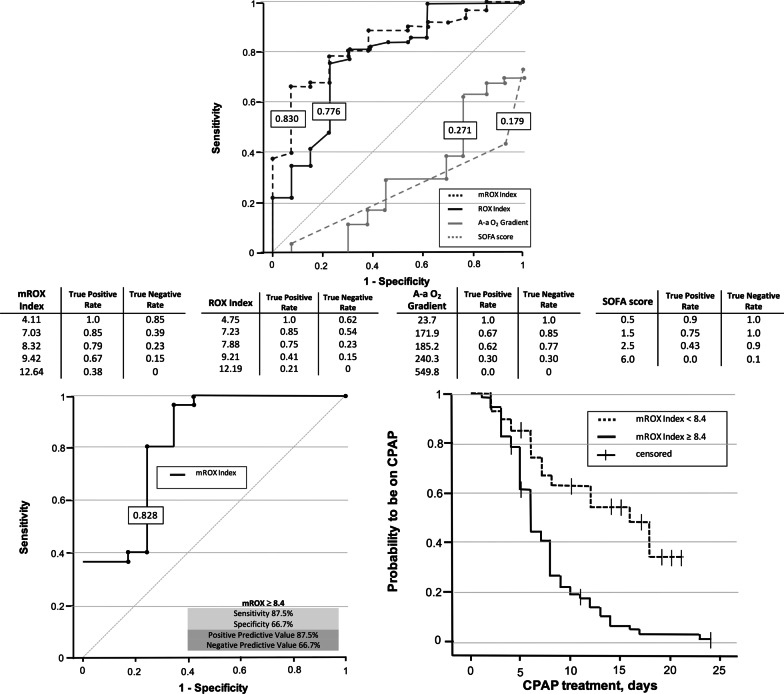
Accuracy and performance of predictors of weaning success from helmet CPAP. Receiver operating characteristic (ROC) curves with areas under the ROC curves showing the performance for each index in predicting weaning success (upper panel). Sensitivity and specificity for each weaning predictor is also reported. The left lower panel shows the performance of the mROX threshold of 8.4 mmHg/bpm in predicting weaning outcome in the validation cohort. The right lower panel illustrates the probability to remain on CPAP during the hospital stay in the pooled population (n = 118) in patients with a mROX index of ≥ or < 8.4. CPAP = continuous positive expiratory pressure

Our data demonstrated that the mROX index, combining non-invasive surrogates of respiratory distress (RR) and gas exchange efficiency (PaO_2_/FiO_2_), was the best predictor of weaning success from CPAP. We observed a relatively low rate of weaning failure, suggesting that weaning attempts tend to be performed late, and reflecting the need for objective and sensitive indicators of weaning preparedness, as for invasive mechanical ventilation [5].

Some limitations need further exploration. First, these thresholds should be tested in randomized clinical trials and compared with standard of care. Second, predictors should be sequentially measured at different time-points during zero-PEEP, to assess their performance variability during the weaning trial and unassisted breathing [2, 6].

In conclusion, the mROX threshold of 8.4 mmHg/bpm appears a sensitive and robust predictor of weaning success from helmet CPAP in patients with COVID-19.

## Data Availability

Individual patient data will be available, upon individual and specific request, to researchers whose proposed use of the data has been approved. Data will be made available request to: pierachille.santus@unimi.it. Data will be provided with investigator support, after approval and after signing a data access agreement. The use of individual patient data outside personal consultation will not be permitted.
